# Integrating Electronic Health Records and Large Language Models for Coarse-to-Fine Hybrid Disease Prediction

**DOI:** 10.34133/hds.0466

**Published:** 2026-06-08

**Authors:** Chutong Wang, Mingda Li, Xuebing Yang, Mengxuan Sun, Wensheng Zhang

**Affiliations:** ^1^ Guangzhou University, Guangzhou, China.; ^2^ State Key Laboratory of Multimodal Artificial Intelligence Systems, Institute of Automation, Chinese Academy of Sciences, Beijing, China.; ^3^ Nankai University, Tianjin, China.

## Abstract

**Background:** Electronic health records (EHRs) have been widely adopted in recent years. However, diagnosis codes in EHRs are often high-dimensional, abstract, and follow a long-tailed distribution, limiting the effectiveness and interpretability of traditional data-driven models for disease prediction. Large language models (LLMs) offer a promising alternative due to their broad knowledge and strong reasoning capabilities, yet their direct application remains challenging due to the vast prediction space and limited domain adaptation. **Methods:** To address these challenges, we propose a coarse-to-fine hybrid disease prediction framework. First, an EHR-driven base prediction module is used to generate coarse predictions. Second, patient information is serialized using a tailored prompt template, and a fine-tuned LLM further assesses the presence of high-probability candidate diseases. Finally, an aggregation module integrates outputs from both modules to yield refined predictions. **Results:** The coarse-to-fine hybrid disease prediction framework consistently outperforms existing non-LLM and LLM-based approaches in disease prediction. Specifically, compared to the second-best baselines, improvements of 1.6% on P@5, 1.1% on P@10, 0.7% on P@20, 0.3% on R@5, 1.6% on R@10, 1.0% on R@20, 1.4% on w-F1, and 1.0% on Jaccard for the eICU Collaborative Research Database and improvements of 2.6% on P@5, 4.3% on P@10, 4.2% on P@20, 4.2% on R@5, 5.9% on R@10, 4.5% on R@20, 6.0% on w-F1, and 2.5% on Jaccard for the Medical Information Mart for Intensive Care IV are achieved, respectively. Further analyses demonstrate the effectiveness of each component of the framework. **Conclusion:** By integrating EHR-driven base prediction with LLM-based assessments in a coarse-to-fine manner, this study provides an effective and practical disease-prediction framework for clinical decision support.

## Introduction

In recent years, electronic health records (EHRs), encompassing demographic information and a sequence of visit records for each patient, have been widely adopted in healthcare systems [1,2]. Serving as a high-quality data resource, EHRs have provided promising opportunities for clinical predictive research, such as mortality prediction [3,4] and medication recommendation [5,6]. Effective predictions are vital for healthcare because they can provide timely health alerts and personalized care plans [7].

**Fig. 1.**
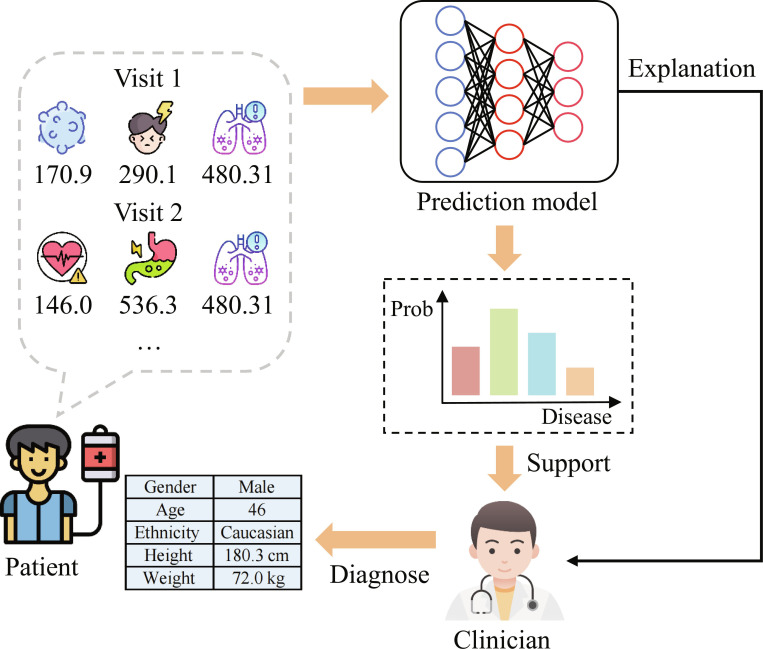
An illustration of a typical clinical decision support system for disease prediction. The collected patient information is input to the disease-prediction model, which analyzes the data and generates predictions for clinicians.

Generally, each visit record includes various health events, where diseases are represented as standard diagnosis codes such as the International Classification of Diseases, 9th revision (ICD-9) and the International Classification of Diseases, 10th revision (ICD-10) [8]. In the disease-prediction community, a typical workflow is leveraging diagnosis codes in historical visits to predict those in the final visit. Figure 1 illustrates a clinical decision support system for disease prediction, with the prediction model serving as the critical part to generate predictions for decision support. To this end, early studies apply traditional machine-learning models, such as Support Vector Machine [9] and XGBoost [10]. In the era of deep learning, deep neural network approaches, such as recurrent neural network (RNN)-based models [11–13] and attention-based models [14–16], have been widely adopted. These approaches learn disease progression patterns primarily by capturing temporal dependencies and co-occurrence relationships among diagnosis codes observed in EHR data. Although such EHR data-driven approaches have achieved promising performance in disease prediction, they still face several problems, including limited reasoning capability and poor interpretability.

Problem 1 (Limited reasoning capability). In EHRs, diagnosis codes are often high-dimensional, abstract, and follow a long-tailed distribution, with a considerable proportion appearing infrequently, which substantially hinders EHR data-driven models from capturing meaningful semantic relationships and causal correlations [17,18]. Consequently, such models often exhibit limited reasoning capability, motivating the need to incorporate broader semantic knowledge and stronger reasoning mechanisms.

Problem 2 (Poor interpretability). Existing EHR-driven models often suffer from poor interpretability and fall into the “black box” paradigm, which undermines clinicians’ trust in model predictions [19,20]. Although some studies attempt to explain predictions by visualizing attention weights or feature importance scores, such explanations are often indirect and difficult for clinicians to interpret. There is an urgent need to provide explicit rationales.

The advent of large language models (LLMs) offers a promising direction to address the aforementioned problems. Pretrained on massive corpora, LLMs possess broad medical knowledge, strong reasoning capability, and the ability to generate intuitive textual rationales [21,22]. Motivated by these advantages, recent studies have begun to explore the integration of LLMs into disease-prediction tasks, which can be broadly categorized into 2 groups. One group leverages LLMs to perform disease prediction in constrained settings, such as specific-disease risk assessment or multiple-choice diagnosis [23–26], while the other group employs off-the-shelf LLMs to encode disease representations or generate disease synonyms for data augmentation, without domain-specific fine-tuning [27,28]. Despite their promising results, effectively integrating LLMs into disease prediction remains challenging:

Challenge 1 (Vast prediction space). Disease prediction involves a large set of diseases to predict. If the full set of diseases is not provided to LLMs as a cue, they may fail to consider all relevant conditions. However, incorporating the entire set is impractical, as it results in excessively long inputs that are difficult for the LLM to process effectively. A straightforward solution is to query LLMs for the presence of each disease individually. However, such an approach incurs prohibitive reasoning costs, unless the candidate disease space can be effectively reduced in advance.

Challenge 2 (Limited domain adaptation). Although LLMs are pretrained on large-scale medical corpora, they may lack specialized clinical expertise and struggle to effectively utilize patient information for inference. Without proper adaptation, LLMs may generate clinically unreliable outputs, such as hallucinations, which limits their practical applicability in clinical settings.

To address the aforementioned challenges, we propose a novel coarse-to-fine hybrid disease prediction framework, termed CFHDP. Unlike most existing approaches, CFHDP adopts a hybrid design that integrates an EHR-driven base prediction module with an LLM in a complementary manner. To adapt the LLM to the disease-prediction domain, we design a tailored prompt template and employ low-rank adaptation (LoRA, Hu et al. [29]) for fine-tuning, addressing Challenge 2. For each patient, the base prediction module first generates a coarse disease prediction, from which high-probability diseases are automatically selected as candidates. The LLM then assesses the presence of each candidate, addressing Challenge 1. Finally, a result aggregation module organically fuses the outputs of both modules, yielding a refined prediction.

Our main contributions are as follows:

• We propose a novel coarse-to-fine hybrid framework CFHDP for disease prediction, which integrates EHR-driven base prediction and LLM-based assessments in a complementary manner. Unlike traditional EHR-driven approaches, our CFHDP simultaneously provides detailed rationales, which can serve as a textual reference for explaining predicted diseases.
• To effectively adapt LLMs for disease prediction, we design a tailored prompt template and adopt LoRA to fine-tune LLMs. Besides, a result aggregation module is introduced to organically fuse the outputs from both modules.
• Extensive experiments conducted on eICU Collaborative Research Database (eICU-CRD) and Medical Information Mart for Intensive Care IV (MIMIC-IV) datasets demonstrate that CFHDP consistently outperforms state-of-the-art baselines on disease prediction. Further analyses validate the effectiveness of each component.

## Methods

### Preliminary

**Definition 1 (EHR data)**. Given an EHR dataset ℰ, each patient record $P^{\left(i\right)}\in ℰ$ consists of a demographic feature set $D^{\left(i\right)}$ and a sequence of visit records $V_{1}^{\left(i\right)},V_{2}^{\left(i\right)},\ldots ,V_{T^{\left(i\right)}}^{\left(i\right)}$. For simplicity, in the following we describe our method for a single patient and omit superscript (*i*). We denote the set of diagnosis codes appearing in ℰ as $C=\left\{c_{1},\;c_{2},\ldots ,\;c_{|C|}\right\}$, where ∣C∣ is the number of unique diagnosis codes. Every visit record consists of several diagnosis codes, i.e., $V_{t}\subseteq C,1\leq t\leq T$.

Task 1 (Disease prediction). We regard each diagnosis code in EHRs as a disease. Given a patient record P, disease prediction aims to use D and the visit records before visit *T* to predict $V_{T}$ by generating a probability vector $\hat{y}\in {\mathbb{R}}^{|C|}$.

### Proposed framework

#### Framework overview

**Fig. 2.**
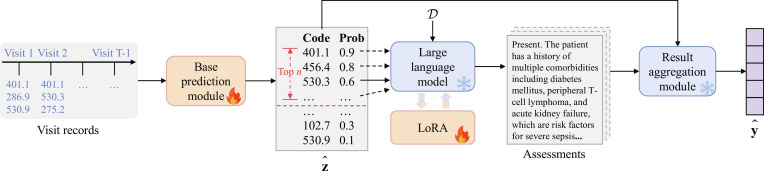
The framework overview of the coarse-to-fine hybrid disease prediction framework (CFHDP). For each patient, the base prediction module generates a coarse prediction $\hat{z}$, from which high-probability diseases are selected as candidates. Then, for each candidate, a prompt is constructed and fed to the large language model (LLM) to produce a presence assessment. The assessments made by the LLM are reorganized and aggregated with $\hat{z}$, resulting in a refined prediction $\hat{y}$. LoRA, low-rank adaptation.

As illustrated in Fig. 2, the proposed CFHDP consists of 3 key modules: an EHR-driven base prediction module, an LLM module, and a result aggregation module. For each patient, the base prediction module learns the patient representation r and generates a coarse disease prediction $\hat{z}$. Based on $\hat{z}$, a subset of high-probability diagnosis codes is selected as candidates, and the LLM module applies a tailored prompt template T to further assess the presence of each candidate. Finally, the result aggregation module combines the LLM assessments with $\hat{z}$, resulting in a refined prediction $\hat{y}$.

#### EHR-driven base prediction module

**Fig. 3.**
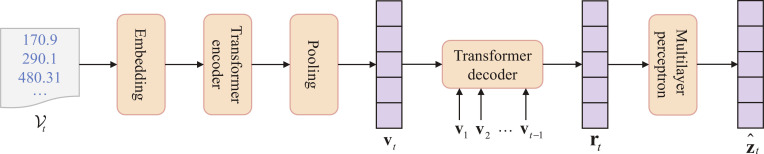
Base prediction module. For visit *t*, $V_{t}$ is used to derive the visit representation $v_{t}$. Then, $v_{t}$ is aggregated with the visit representations from earlier visits to form the patient representation $r_{t}$, which is used to generate the prediction ${\hat{z}}_{t}$ for visit *t*.

The EHR-driven base prediction module is shown in Fig. 3. To fully exploit the EHR dataset, we adopt an autoregressive training strategy, i.e., sequentially predicting the diagnosis codes from visit 2 to visit *T*. For visit *t* (1≤t≤T−1), each diagnosis code in $V_{t}$ is first projected into a $d_{e}$-dimensional vector through an embedding layer, forming $E_{t}\in {\mathbb{R}}^{|V_{t}|\times d_{e}}$:

$$E_{t}=\text{Embedding}\left(V_{t}\right).$$ (1)

Next, $E_{t}$ is fed into a Transformer encoder [30] to capture correlations among diagnosis codes. The encoded representations are then passed through a pooling layer followed by a linear layer to obtain the visit representation $v_{t}\in {\mathbb{R}}^{d_{r}}$:

$$\begin{array}{cc} & E_{t}^{\prime }=\text{TransformerEncoder}\left(E_{t}\right),\\ & v_{t}=\text{Linear}\;\left(\text{Pooling}\left(E_{t}^{\prime }\right)\right). \end{array}$$ (2)

To incorporate longitudinal information, we employ a Transformer decoder to aggregate visit representations up to visit *t*, resulting in the patient representation $r_{t}\in {\mathbb{R}}^{d_{r}}$:

$$r_{t}=\text{TransformerDecoder}\left(v_{t},\;\left\{v_{1},\;v_{2},\;\ldots ,\;v_{t-1}\right\}\right),$$ (3)

and further use $r_{t}$ to predict diagnosis codes at visit t+1:

$${\hat{z}}_{t}=\sigma \left(\text{Linear}\left(r_{t}\right)\right),$$ (4)

where ${\hat{z}}_{t}\in {\mathbb{R}}^{|C|}$ and $\sigma \left(\cdot \right)$ denote the sigmoid function.

#### LLM for disease prediction

After deriving the coarse prediction $\hat{z}={\hat{z}}_{T-1}$ from the base prediction module, we employ an LLM to assess the presence of diagnosis codes individually. Since the actual number of diagnosis codes at visit *T* is much smaller than ∣C∣, we restrict LLM inference to a subset of high-probability codes to reduce cost. Specifically, the LLM is queried for a diagnosis code $c_{i}$ only if its predicted probability $p_{i}$ is among the top *n* in $\hat{z}$, where *n* is a hyperparameter. These *n* diagnosis codes are treated as candidate codes, forming a candidate set $\begin{array}{c} S=\left\{\left(c_{i},\;p_{i}\right)|p_{i}\;\text{is among the}\;\mathrm{top}\;n\;\text{values in}\;\hat{z}\right\} \end{array}$.

**Fig. 4.**
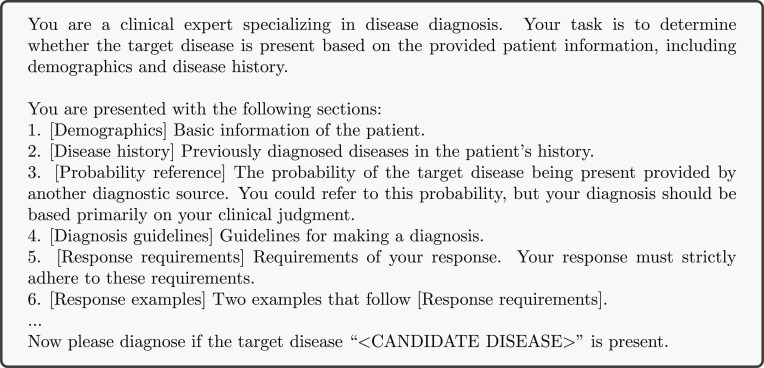
Abridged prompt template for LLM assessments. Text enclosed in angle brackets will be instantiated with actual values.

To bridge the gap between structured patient data and unstructured textual input required by the LLM, we utilize public resources to convert diagnosis codes into their textual descriptions (e.g., using Wikipedia code-to-text mappings [https://en.wikipedia.org/wiki/List_of_ICD-9_codes] for ICD-9 codes) and incorporate the patient information in P into our tailored prompt template T. The abridged version of T is shown in Fig. 4 (see Fig. S1 for the complete version), where the text enclosed in angle brackets will be instantiated with actual values. The template includes a task definition and several sections. In particular, the demographics section provides basic patient information, such as age and gender. The disease history is constructed from diseases appearing in previous visit records. The probability reference is provided by the base prediction module, while diagnostic guidelines and response examples are used to guide the LLM toward rational assessments.

Although LLMs exhibit strong reasoning capabilities, their performance in healthcare can be limited without task-specific adaptation. To adapt the LLM for disease prediction, we employ LoRA for fine-tuning, where only low-rank matrices are updated while the original LLM’s weights remain frozen. The trainable parameters are denoted as ${\left\{A_{i},\;B_{i}\right\}}_{i=1}^{L}$, where *L* is the number of layers equipped with LoRA modules. In real-world EHR settings, ground-truth explanatory annotations are typically difficult to obtain at scale. Therefore, during fine-tuning, we modify the response requirements in T to constrain the model output to a binary assessment conclusion, i.e., “Present” or “Absent”, with supervision provided solely by the ground-truth presence of the corresponding diagnosis codes.

At inference time, in addition to producing a binary conclusion, the LLM is also prompted to generate a corresponding rationale for each candidate code, following the response requirements specified in T. Such rationales leverage the LLM’s semantic representation ability acquired during pretraining and are intended to reflect the reasoning underlying the predicted conclusions.

#### Result aggregation

To maximize the complementary strengths of the base prediction module and the LLM, we design a result aggregation module that assigns code-specific weights to their outputs. Specifically, for each diagnosis code $c_{i}$, we compute the F1 scores of the base prediction module and the LLM on the training and validation data. If $c_{i}$ never appears in S, we assign an F1 score of zero to the LLM for this code. This yields 2 F1 score vectors: $f_{p}\in {\mathbb{R}}^{|C|}$ for the base prediction module and $f_{l}\in {\mathbb{R}}^{|C|}$ for the LLM.

Based on these F1 scores, we calculate their weights $w_{b},w_{l}\in {\mathbb{R}}^{|C|}$, which are applied during inference. If both F1 scores for a given code are zero, the weights are computed using the overall F1 scores across all codes:

$$\begin{array}{c} w_{b}\left[i\right]=\left\{\begin{array}{ccc} \frac{f_{p}\left[i\right]}{f_{p}\left[i\right]+f_{l}\left[i\right]}, & f_{p}\left[i\right]+f_{l}\left[i\right]\neq 0, & \\ \frac{\sum_{i}^{|C|}f_{p}\left[i\right]}{\sum_{i}^{|C|}f_{p}\left[i\right]+\sum_{i}^{|C|}f_{l}\left[i\right]}, & \text{otherwise}, \end{array}\right. \end{array}$$ (5)

$$w_{l}\left[i\right]=1-w_{b}\left[i\right].$$ (6)

For each candidate code $c_{i}\in S$, the assessment conclusion produced by the LLM, i.e., “Present” or “Absent”, is converted into a numerical value $v_{i}\in \left\{0,\;1\right\}$. Then, the results from the 2 modules are aggregated using $w_{b}$ and $w_{l}$ to produce the refined prediction $\hat{y}\in {\mathbb{R}}^{|C|}$:

$$\hat{y}\left[i\right]=\left\{\begin{array}{ccc} w_{b}\left[i\right]\hat{z}\left[i\right]+w_{l}\left[i\right]v_{i}, & \left(c_{i},\;p_{i}\right)\in S, & \\ \hat{z}\left[i\right], & \text{otherwise}. & \end{array}\right.$$ (7)

### Train and inference

As disease prediction is a multilabel binary classification task, the binary cross-entropy loss is used, where y denotes the ground truth:

$$L=-\left(y\;\text{log}\;\hat{y}+\left(1-y\right)\text{log}\left(1-\hat{y}\right)\right).$$ (8)

The proposed CFHDP employs a 2-stage training procedure. In stage 1, the base prediction module is trained using Eq. (8). In stage 2, the base prediction module is frozen and used to generate the candidate set S. Then, for each candidate, a prompt is constructed to fine-tune the LLM using LoRA, where only the low-rank matrices are updated. During inference, $w_{b}$ and $w_{l}$ are computed. After the results of the base prediction module and the LLM are generated, the refined prediction is obtained according to $w_{b}$ and $w_{l}$. The procedure of training and inference is summarized in Algorithm 1.


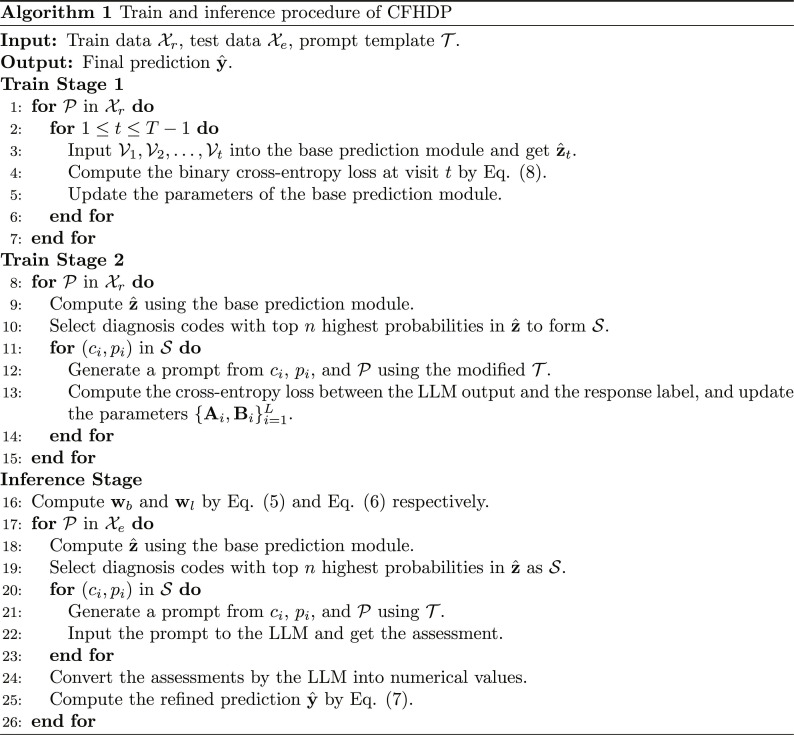


### Dataset

To evaluate the effectiveness of the CFHDP, we conduct experiments on eICU-CRD [31], a multicenter dataset covering 139,367 patient records from 2014 to 2015 across the United States, and MIMIC-IV [8], a single-center dataset containing 73,181 intensive-care-unit patient records from 2008 to 2019.

We extract ICD-9 diagnosis codes from each patient record for disease prediction. Patients with fewer than 2 visits and visits without any diagnosis codes are excluded. As a result, we obtain 8,209 patient records with 17,594 visits for eICU-CRD and 6,561 patient records with 27,497 visits for MIMIC-IV. We use the diagnosis codes in the last visit as the label and treat those from earlier visits as features for each patient. Furthermore, we extract 5 demographic features from each patient record for LLM prompting, including gender, age, ethnicity, height, and weight. The statistics of the preprocessed datasets are presented in Table 1.

**Table 1.** Statistics of the preprocessed datasets

Item	eICU-CRD	MIMIC-IV
No. of patients	8,209	6,561
No. of visits	17,594	27,497
Max. no. of visits per patient	7	50
Avg. no. of visits per patient	2.14	4.19
No. of diagnosis codes	656	4,798
Max. no. of codes per visit	53	39
Avg. no. of codes per visit	4.23	11.62
No. of demographic features	5	5

eICU-CRD, eICU Collaborative Research Database; MIMIC-IV, Medical Information Mart for Intensive Care IV

### Baselines

In the experiments, we compare the proposed CFHDP with several state-of-the-art disease-prediction models:

• RETAIN [11]. An RNN-based network. After reversing the order of the visit sequence, 2 RNNs are employed to encode visit-level weight and variable-level weight, respectively, to measure the importance of a variable to the target disease.
• GRAM [14]. A hybrid model with a medical ontology graph, which uses the medical ontology to learn the embeddings of diagnosis codes and their ancestors, and then incorporates an attention mechanism to aggregate embeddings for prediction.
• Timeline [32]. An RNN-based model with attention modules. After summing the embeddings of the diagnosis codes as visit representations, an RNN is employed for prediction.
• HiTANet [33]. A hierarchical time-aware attention network that embeds the irregular time information to vectors and uses attention modules to encode time information at local and global levels.
• Chet [34]. A context-aware learning framework using transition functions on dynamic disease graphs. The disease graphs are divided to different views based on the co-occurrence relationship for prediction.
• CATNet [13]. A cross-event attention-based network, which aggregates different event information via attention modules and uses a task-aware attention for prediction.
• Sherbet [35]. A self-supervised graph learning framework. The model first learns the representations of the diagnosis codes using the ontology structure and co-occurrence information. Then, a historical hierarchy prediction task is used to pretrain the encoder modules before inference.
• BioDynGrap [36]. A framework that uses dynamic graph encoding with temporal reasoning to capture the evolving nature of disease progression.
• IICL [37]. An architecture that integrates graph modeling and contrastive learning to capture explicit and implicit disease relationships, enhancing patient representations.
• ADRL [38]. An adaptive disease representation learning framework, which dynamically optimizes disease relationships by performing self-supervised perturbations and integrates personalized feature learning for prediction.
• LLM-DG [27]. A framework that leverages an LLM to encode patient and disease representations and jointly models interpatient and intrapatient relationships for prediction.

Additionally, we include HuatuoGPT-o1-8B (https://huggingface.co/FreedomIntelligence/HuatuoGPT-o1-8B), MedGemma-27B (https://huggingface.co/google/medgemma-27b-text-it), GPT-5.2 (https://openai.com/index/introducing-gpt-5-2/), and fine-tuned Qwen3-8B (https://huggingface.co/Qwen/Qwen3-8B) (abbr., ftd. Qwen3-8B) as end-to-end LLM baselines. Among them, HuatuoGPT-o1-8B and MedGemma-27B are medical LLMs pretrained on large-scale medical corpora, while GPT-5.2 is a closed-source LLM that has demonstrated superior reasoning capability [39]. We fine-tune Qwen3-8B using the same training samples as CFHDP for disease prediction. For these baselines, patient information is serialized into prompts, and the models directly predict the set of diseases for a patient in an end-to-end manner. Since the generated diseases may not exactly correspond to the diagnosis codes in C, we perform a semantic matching procedure for evaluation. Specifically, we encode both the generated diseases and the textual descriptions of all codes in C using the BGE-M3 embedding model (https://huggingface.co/BAAI/bge-m3). For each generated disease, the diagnosis code with the highest cosine similarity above 0.7 is selected as the predicted code; otherwise, the disease is treated as unmatched.

### Evaluation metrics

We use precision@k (abbr., P@k) [40], recall@k (abbr., R@k) [41], w-F1 [32], and Jaccard similarity score (abbr., Jaccard) [42] as evaluation metrics for disease prediction. P@k measures the average ratio of correct diagnosis codes in the top *k* predictions by $\min\left(k,|V_{T}|\right)$ for each patient. R@k measures the average ratio of correct diagnosis codes in the top *k* predictions by $|V_{T}|$ for each patient. w-F1 is the weighted average of F1 scores for all diagnosis codes. Jaccard is the ratio of the intersection set between $V_{T}$ and the set of codes with the predicted probabilities greater than 0.5, divided by their union set. Among them, P@k and R@k are ranking based and less sensitive to exact probability values, focusing instead on whether the correct diagnosis codes appear in the top-ranked predictions. In contrast, w-F1 and Jaccard are threshold based and sensitive to whether the predicted probabilities exceed the 0.5 threshold. These metrics assess disease-prediction performance from different perspectives.

## Results

### Implementation details

In the experiments, patients are randomly split into training, validation, and test sets with a ratio of 8:1:1. All experiments are conducted on a CentOS 7.9 server equipped with 3 NVIDIA A40 GPUs. Our proposed CFHDP is implemented using PyTorch 2.4 with CUDA 12.1.

For training the base prediction module, we use the AdamW optimizer with a learning rate of 1 × 10^−4^ and train the model for 100 epochs. Both the embedding dimension d_e_ and the representation dimension d_r_ are set to 256.

For the LLM module, Qwen3-8B is adopted as the default LLM backbone in the main experiments. The number of top-probability diagnosis codes selected for further LLM assessment is set to 20. During fine-tuning, LoRA is applied to the layers “q_proj”, “v_proj”, “k_proj”, “o_proj”, “gate_proj”, “up_proj”, and “down_proj”. The LoRA rank is set to 8, with a LoRA alpha of 32 and a learning rate of 2 × 10^−5^. The LLM is fine-tuned for up to 2 epochs with early stopping and a patience of 2. The fine-tuning process requires approximately 94 GB of GPU memory.

During inference, the maximum output token length is set to 512. The top-*P* value is 0.8 and the temperature is 0.2. Inference requires approximately 16 GB GPU memory, with an average latency of about 5 s per disease-presence assessment.

### Results

The performance comparison between the proposed CFHDP and baselines is reported in Table 2 and Table 3. We perform 5 randomized runs and report “mean_standard deviation_” for each approach. We select *k* = [5,10,20] to calculate P@k and R@k because the average numbers of diagnosis codes per visit are 4.23 and 11.62 in eICU-CRD and MIMIC-IV, respectively.

**Table 2.** Performance of disease prediction compared to baselines on the eICU-CRD

Model	P@5	P@10	P@20	R@5	R@10	R@20	w-F1	Jaccard
Non-LLM approaches								
Retain	$80.10_{0.13}$	$81.62_{0.07}$	$85.59_{0.14}$	$73.71_{0.11}$	$80.63_{0.10}$	$84.82_{0.12}$	$61.97_{0.14}$	$49.28_{0.17}$
GRAM	$72.79_{0.13}$	$75.36_{0.11}$	$81.92_{0.09}$	$64.06_{0.11}$	$73.58_{0.12}$	$80.85_{0.12}$	$57.92_{0.13}$	$17.33_{0.18}$
Timeline	$78.54_{0.21}$	$79.70_{0.19}$	$84.69_{0.22}$	$71.96_{0.21}$	$78.59_{0.26}$	$83.92_{0.29}$	$65.11_{0.13}$	$61.74_{0.27}$
HiTANet	$80.50_{0.22}$	$81.81_{0.18}$	$85.49_{0.14}$	$73.58_{0.11}$	$80.83_{0.12}$	$84.72_{0.11}$	$67.17_{0.13}$	$64.64_{0.18}$
Chet	$80.03_{0.21}$	$83.54_{0.16}$	$86.42_{0.15}$	$73.97_{0.18}$	$81.72_{0.16}$	$85.96_{0.14}$	$64.60_{0.14}$	$63.14_{0.17}$
CATNet	$81.70_{0.14}$	$83.43_{0.20}$	$86.90_{0.12}$	$75.43_{0.19}$	$82.20_{0.13}$	$86.42_{0.11}$	$68.64_{0.13}$	$64.94_{0.12}$
Sherbet	$82.62_{0.12}$	$83.60_{0.13}$	$87.04_{0.12}$	$76.69_{0.13}$	$82.43_{0.10}$	$86.77_{0.13}$	$67.81_{0.18}$	$64.34_{0.14}$
BioDynGrap	$81.83_{0.18}$	$83.47_{0.21}$	$86.71_{0.17}$	$76.23_{0.14}$	$81.42_{0.19}$	$86.51_{0.16}$	$67.76_{0.12}$	$63.44_{0.14}$
IICL	$82.19_{0.20}$	$83.28_{0.19}$	$86.88_{0.18}$	$76.54_{0.17}$	$81.53_{0.14}$	$86.73_{0.15}$	$68.52_{0.13}$	$64.67_{0.16}$
ADRL	$82.43_{0.19}$	$83.42_{0.17}$	$87.19_{0.16}$	$76.43_{0.15}$	$82.02_{0.16}$	$87.14_{0.18}$	$68.87_{0.14}$	$64.81_{0.13}$
LLM-based approaches								
HuatuoGPT-o1-8B	$55.53_{0.13}$	$52.72_{0.09}$	$52.12_{0.11}$	$50.77_{0.07}$	$51.74_{0.12}$	$51.89_{0.06}$	$53.74_{0.11}$	$44.83_{0.10}$
MedGemma-27B	$76.54_{0.08}$	$74.91_{0.13}$	$75.13_{0.11}$	$70.48_{0.05}$	$73.62_{0.13}$	$74.87_{0.09}$	$68.84_{0.10}$	$66.42_{0.06}$
GPT-5.2	$75.84_{0.06}$	$73.52_{0.07}$	$73.44_{0.05}$	$70.03_{0.07}$	$72.22_{0.04}$	$72.18_{0.05}$	$69.06_{0.04}$	$65.24_{0.09}$
ftd. Qwen3-8B	$82.43_{0.06}$	$80.84_{0.04}$	$80.47_{0.08}$	$76.24_{0.05}$	$79.46_{0.7}$	$80.24_{0.08}$	${\underline{69.84}}_{0.06}$	${\underline{68.18}}_{0.09}$
LLM-DG	${\underline{82.82}}_{0.15}$	${\underline{83.83}}_{0.07}$	${\underline{87.72}}_{0.13}$	${\underline{77.05}}_{0.11}$	${\underline{82.61}}_{0.06}$	${\underline{87.43}}_{0.14}$	$69.22_{0.09}$	$65.93_{0.05}$
CFHDP	$84.13_{0.09}^{*}$	$84.78_{0.12}^{*}$	$88.37_{0.16}^{*}$	$77.26_{0.04}^{*}$	$83.94_{0.10}^{*}$	$88.34_{0.08}^{*}$	$70.82_{0.14}^{*}$	$68.84_{0.06}^{*}$

The best result is in bold, and the second-best result is underlined. Subscripts denote standard deviations. * denotes the coarse-to-fine hybrid disease prediction framework (CFHDP) is statistically significant compared with all baselines (all *t*-test *P* values < 0.05). LLM, large language model.

**Table 3.** Performance of disease prediction compared to baselines on the MIMIC-IV

Model	P@5	P@10	P@20	R@5	R@10	R@20	w-F1	Jaccard
Non-LLM approaches								
Retain	$46.09_{0.21}$	$36.30_{0.12}$	$37.21_{0.18}$	$20.17_{0.13}$	$28.72_{0.11}$	$36.31_{0.13}$	$23.93_{0.14}$	$11.49_{0.13}$
GRAM	$43.44_{0.22}$	$37.73_{0.18}$	$41.13_{0.17}$	$23.06_{0.19}$	$31.17_{0.14}$	$40.37_{0.13}$	$20.72_{0.12}$	$9.94_{0.20}$
Timeline	$46.58_{0.19}$	$35.63_{0.13}$	$35.64_{0.20}$	$19.87_{0.12}$	$27.81_{0.11}$	$34.59_{0.14}$	$23.91_{0.11}$	$15.28_{0.13}$
HiTANet	$50.98_{0.21}$	$39.87_{0.13}$	$39.64_{0.14}$	$21.86_{0.14}$	$30.04_{0.12}$	$38.63_{0.12}$	$26.32_{0.11}$	$17.62_{0.13}$
Chet	$49.29_{0.13}$	$39.07_{0.14}$	$40.17_{0.14}$	$23.17_{0.13}$	$31.08_{0.16}$	$39.04_{0.17}$	$26.09_{0.17}$	$15.13_{0.18}$
CATNet	$50.31_{0.19}$	$40.88_{0.21}$	$41.39_{0.22}$	$23.34_{0.21}$	$30.97_{0.21}$	$40.07_{0.14}$	$26.86_{0.13}$	$17.73_{0.12}$
Sherbet	$50.87_{0.18}$	$41.14_{0.19}$	$40.76_{0.18}$	$23.28_{0.19}$	$31.73_{0.20}$	$39.63_{0.13}$	$26.45_{0.14}$	$16.54_{0.11}$
BioDynGrap	$50.26_{0.16}$	$41.17_{0.15}$	$40.79_{0.17}$	$23.06_{0.18}$	$31.02_{0.13}$	$39.51_{0.19}$	$27.24_{0.14}$	$16.74_{0.16}$
IICL	$50.52_{0.17}$	$40.68_{0.14}$	$41.00_{0.16}$	$23.26_{0.15}$	$30.78_{0.15}$	$39.93_{0.19}$	$27.29_{0.14}$	$17.95_{0.17}$
ADRL	$50.95_{0.19}$	$41.36_{0.18}$	$41.32_{0.20}$	$23.49_{0.16}$	$31.60_{0.13}$	$40.25_{0.20}$	$27.67_{0.14}$	$18.33_{0.15}$
LLM-based approaches								
HuatuoGPT-o1-8B	$24.04_{0.08}$	$22.13_{0.08}$	$21.97_{0.05}$	$11.36_{0.06}$	$18.32_{0.10}$	$21.46_{0.10}$	$21.34_{0.07}$	$14.04_{0.11}$
MedGemma-27B	$28.63_{0.11}$	$27.74_{0.06}$	$32.78_{0.07}$	$13.63_{0.09}$	$23.07_{0.06}$	$32.14_{0.08}$	$27.06_{0.05}$	$19.14_{0.04}$
GPT-5.2	$31.27_{0.07}$	$28.96_{0.05}$	$32.93_{0.05}$	$14.44_{0.06}$	$23.86_{0.04}$	$32.16_{0.03}$	$27.17_{0.05}$	$20.02_{0.06}$
ftd. Qwen3-8B	$34.06_{0.10}$	$30.84_{0.04}$	$35.17_{0.10}$	$15.27_{0.08}$	$25.26_{0.11}$	$34.44_{0.08}$	${\underline{28.34}}_{0.05}$	${\underline{20.42}}_{0.07}$
LLM-DG	${\underline{51.19}}_{0.16}$	${\underline{42.04}}_{0.13}$	${\underline{41.69}}_{0.15}$	${\underline{23.74}}_{0.11}$	${\underline{31.70}}_{0.06}$	${\underline{40.56}}_{0.14}$	$28.16_{0.09}$	$19.72_{0.12}$
CFHDP	$52.53_{0.07}^{*}$	$43.86_{0.07}^{*}$	$43.44_{0.10}^{*}$	$24.74_{0.05}^{*}$	$33.56_{0.11}^{*}$	$42.37_{0.08}^{*}$	$30.03_{0.04}^{*}$	$20.94_{0.06}^{*}$

The best result is in bold, and the second-best result is underlined. Subscripts denote standard deviations. * denotes CFHDP is statistically significant compared with all baselines (all *t*-test *P* values < 0.05).

We observe that CFHDP outperforms all baselines across all metrics. In particular, compared to the second-best performance, CFHDP achieves improvements of 1.6% on P@5, 1.1% on P@10, 0.7% on P@20, 0.3% on R@5, 1.6% on R@10, 1.0% on R@20, 1.4% on w-F1, and 1.0% on Jaccard for eICU-CRD and improvements of 2.6% on P@5, 4.3% on P@10, 4.2% on P@20, 4.2% on R@5, 5.9% on R@10, 4.5% on R@20, 6.0% on w-F1, and 2.5% on Jaccard for MIMIC-IV. These consistent gains on both rank-based and threshold-based metrics highlight the CFHDP’s ability to provide precise predictions, which are crucial for real-world clinical decision-making.

The second-best performance across most metrics is achieved by LLM-DG, as it enriches code representations with LLM-based semantics. However, LLM-DG relies on frozen LLM features, which limits its ability to uncover certain disease correlations in EHRs. In contrast, the fine-tuned Qwen3-8B achieves the second-best performance on w-F1 and Jaccard, demonstrating the value of domain adaptation for disease prediction.

Overall, end-to-end LLMs, including large-scale MedGemma-27B and closed-source GPT-5.2, underperform non-LLM baselines on most metrics. This is primarily because they lack an explicit prediction space as a cue. In contrast, non-LLM baselines are trained with a fixed output layer that predicts probabilities for all diseases. Notably, unlike end-to-end LLM baselines, the LLM in our CFHDP is used solely to assess a small set of candidate diseases, framing the task as binary classification and enabling more focused reasoning.

The performance of all approaches is obviously better on eICU-CRD than on MIMIC-IV. This discrepancy can be attributed to the fact that MIMIC-IV involves a larger prediction space than eICU-CRD. Additionally, on eICU-CRD, the performance on P@k (*k* = 5, 10, and 20) consistently improves as *k* increases for all approaches. However, this trend is not always observed on MIMIC-IV, mainly due to the definition of P@k and the different average numbers of diagnosis codes per visit across the 2 datasets.

### Ablation study

To validate the contribution of each component of CFHDP to disease prediction, we conduct an ablation study on eICU-CRD and MIMIC-IV. The results are reported in Table 4 and Table 5, respectively.

**Table 4.** Ablation study on eICU-CRD for disease prediction

Model	P@5	P@10	P@20	R@5	R@10	R@20	w-F1	Jaccard
Only base	82.05	83.02	86.41	74.78	81.95	86.65	68.83	65.13
Only LLM	82.43	80.84	80.47	76.24	79.46	80.24	69.84	68.18
w/o ft	79.85	80.33	87.00	72.72	79.26	87.19	66.93	50.89
wa	82.97	84.04	87.41	75.30	83.14	87.61	70.06	68.44
CFHDP	**84.13**	**84.78**	**88.37**	**77.26**	**83.94**	**88.34**	**70.82**	**68.84**

The best result is in bold. “w/o” denotes without, “ft” denotes fine-tuning, and “wa” denotes weighted average.

**Table 5.** Ablation study on the MIMIC-IV for disease prediction

Model	P@5	P@10	P@20	R@5	R@10	R@20	w-F1	Jaccard
Only base	50.64	41.13	41.79	23.09	31.79	40.50	27.44	17.22
Only LLM	34.06	30.84	35.17	15.27	25.26	34.44	28.34	20.42
w/o ft	50.87	40.47	42.45	23.01	31.08	41.27	27.10	17.00
wa	51.66	42.45	42.89	23.88	32.36	41.48	28.80	20.55
CFHDP	**52.53**	**43.86**	**43.44**	**24.74**	**33.56**	**42.37**	**30.03**	**20.94**

The best result is in bold. “w/o” denotes without, “ft” denotes fine-tuning, and “wa” denotes weighted average.

#### Effectiveness of the coarse-to-fine hybrid design

We include 2 variants “only base” and “only LLM”. In “only base”, predictions are generated solely by the EHR-driven base prediction module without invoking the LLM. For “only LLM”, we directly adopt the fine-tuned Qwen3-8B baseline introduced in the “Baselines” section, where the LLM performs disease prediction in an end-to-end manner. Note that its performance on eICU-CRD and MIMIC-IV is identical to that reported in Table 2 and Table 3, respectively, ensuring experimental consistency. As shown in Table 4 and Table 5, the 2 variants exhibit strengths on different metrics, indicating their complementary roles in disease prediction. Although both variants achieve competitive performance individually, they consistently underperform CFHDP across all metrics on both datasets. This validates the effectiveness of the proposed coarse-to-fine hybrid design, which leverages the base prediction module to generate coarse predictions and then utilizes the LLM to perform focused presence assessments for high-probability candidates.

#### Effectiveness of LLM fine-tuning

We construct a variant “w/o ft”. Different from CFHDP, the LLM is directly used for presence assessments without fine-tuning. The results show that removing fine-tuning leads to consistent performance degradation. Notably, “w/o ft” even performs worse than “only base” on several metrics, suggesting that incorporating an unadapted LLM may introduce noise or unreliable assessments. This observation highlights that task-specific fine-tuning is essential for adapting the LLM to the disease-prediction task.

#### Effectiveness of the result aggregation module

We implement a variant “wa”, where the prediction for each candidate code is obtained by applying a globally tuned weighted average to the outputs of the base module and the LLM. Despite tuning the weights to achieve the best performance on each dataset, this variant still underperforms CFHDP (refer to the “Module trade-off” section for details). This suggests that uniform fusion is insufficient to fully exploit the complementary strengths of the 2 modules. In contrast, our result aggregation module learns code-specific weights, allowing adaptive module selection at the disease level and leading to more effective predictions.

### Module trade-off

**Fig. 5.**
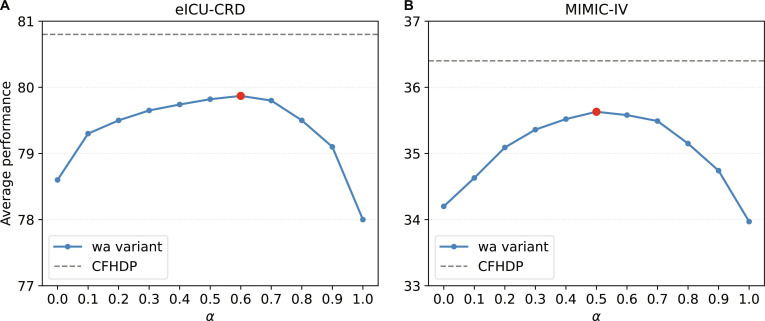
Average performance of the CFHDP and the “wa” variant over 8 metrics under different *α*. eICU-CRD, eICU Collaborative Research Database; MIMIC-IV, Medical Information Mart for Intensive Care IV.

To quantitatively analyze the trade-off between the base prediction module and the LLM, we introduce a global weighting parameter $\alpha \in \left[0,\;1\right]$ in the “wa” variant, where $w_{l}\left[i\right]$ is set to *α* for each candidate code $c_{i}$. By varying *α*, we adjust the relative contributions of the 2 modules. Figure 5 illustrates the average performance over 8 evaluation metrics under different values of *α*. The blue solid curve represents the “wa” variant, while the gray dashed line denotes CFHDP; the best-performing “wa” is highlighted in red. It can be observed that the performance of “wa” improves as α increases from 0 to 0.6 on eICU-CRD (to 0.5 on MIMIC-IV) and degrades thereafter. This pattern highlights the indispensability of both modules. Furthermore, across the entire range of *α*, the “wa” variant consistently underperforms the CFHDP, validating the effectiveness of the result aggregation module in code-specific weight assignment.

### Hyperparameter analysis

**Fig. 6.**
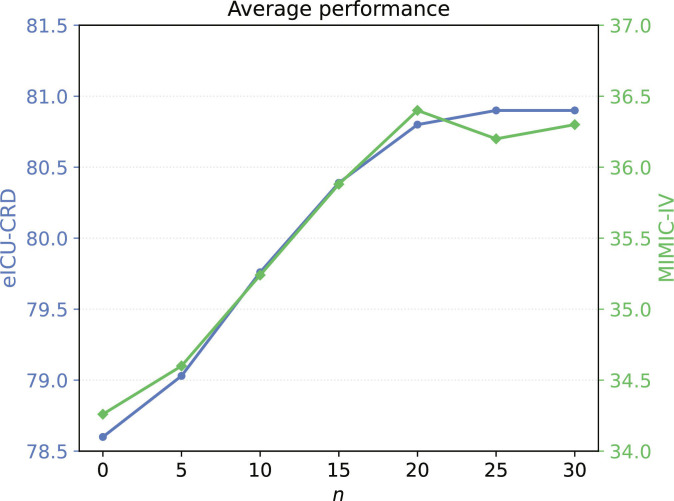
Average performance of the CFHDP and the “wa” variant over 8 metrics under different *n*.

To investigate the choice of the hyperparameter *n* introduced in the “LLM for disease prediction” section, we analyze the average performance of the CFHDP over 8 metrics under different values of *n*. As shown in Fig. 6, on both the eICU-CRD and MIMIC-IV, the overall performance consistently improves as *n* increases from 0 to 20.

Notably, the performance gain from *n* = 0 to *n* = 5 is less pronounced than that from *n* = 5 to *n* = 20. This is primarily because the top-5 diseases are associated with substantially higher predicted probabilities, reflecting lower predictive uncertainty and more reliable predictions. As a result, there is limited room for further improvement by the LLM.

In contrast, the diseases ranked from 6 to 20 generally correspond to lower confidence predictions produced by the base prediction module. In this range, the LLM plays a more critical role by performing complementary assessments, leading to more substantial performance improvements.

When *n* is further increased beyond 20, performance continues to improve slightly on the eICU-CRD, whereas a degradation is observed on the MIMIC-IV, particularly at *n* = 25. This decline is likely due to the inclusion of low-probability diseases for assessment when the available patient information is insufficient, causing the LLM to introduce additional noise through speculative decisions.

Based on these observations, we set *n* = 20 in experiments to balance performance gains and efficiency.

### Module variation

The CFHDP is designed to be modular, allowing flexibility in the choice of both the base prediction module and the LLM module. To investigate the impact of different choices on CFHDP’s performance, we implement 5 variants on the eICU-CRD and MIMIC-IV. The results are reported in Table 6 and Table 7, respectively.

**Table 6.** Module variation on the eICU-CRD for disease prediction

Module	Backbone	P@5	P@10	P@20	R@5	R@10	R@20	w-F1	Jaccard
bm	XGBoost	83.05	83.90	87.66	76.25	83.11	87.05	65.72	58.59
	RETAIN	82.54	83.49	87.60	75.94	83.20	87.08	65.33	56.26
LLM	Llama-3.1-8B	83.62	84.61	88.36	77.31	83.34	87.95	70.25	67.58
	DeepSeek-LLM-7B	83.92	**85.07**	88.19	77.71	83.79	87.84	70.23	66.19
	GLM-4-9B	83.68	84.79	88.29	**78.11**	83.39	88.02	70.38	68.34
	Origin	84.13	84.78	88.37	77.26	83.94	88.34	70.82	**68.84**

“bm” denotes base prediction module.

**Table 7.** Module variation on the MIMIC-IV for disease prediction

Module	Backbone	P@5	P@10	P@20	R@5	R@10	R@20	w-F1	Jaccard
bm	XGBoost	48.89	41.46	40.41	22.05	31.45	38.86	28.09	15.98
	RETAIN	48.20	41.01	40.53	21.97	31.10	38.80	27.82	15.51
LLM	Llama-3.1-8B	51.60	42.79	42.42	24.16	32.46	41.28	28.83	20.54
	DeepSeek-LLM-7B	51.86	42.93	42.62	24.48	32.95	41.47	28.54	20.33
	GLM-4-9B	52.27	43.05	42.43	24.52	32.67	41.19	29.07	**21.33**
	Origin	52.53	43.86	43.44	24.74	33.56	42.37	30.03	20.94

“bm” denotes base prediction module.

#### Variation of the base prediction module

We replace the base prediction module with XGBoost and RETAIN, respectively. This substitution leads to consistent performance degradation across all metrics. This may be attributed to the limitations of both the statistical module and the RNN-based module in capturing the complex correlations among diagnosis codes in EHRs. In contrast, our proposed module leverages attention mechanisms to effectively model these dependencies and generate richer patient representations, resulting in more accurate predictions for subsequent stages.

#### Variation of the LLM module

We replace the original LLM backbone in the CFHDP, i.e., Qwen3-8B, with Llama-3.1-8B (https://huggingface.co/meta-llama/Meta-Llama-3.1-8B-Instruct), DeepSeek-LLM-7B (https://huggingface.co/deepseek-ai/deepseek-llm-7b-chat), and GLM-4-9B (https://huggingface.co/zai-org/glm-4-9b-chat), respectively. It can be found that the replacements improve performance on certain metrics, while our original CFHDP achieves the best performance across most metrics. The observed discrepancies may stem from differences in the pretraining data used for these LLMs. In practical clinical applications, the choice of LLM backbone can be adaptively selected according to the specific metrics of interest.

### Prompt variation

**Fig. 7.**
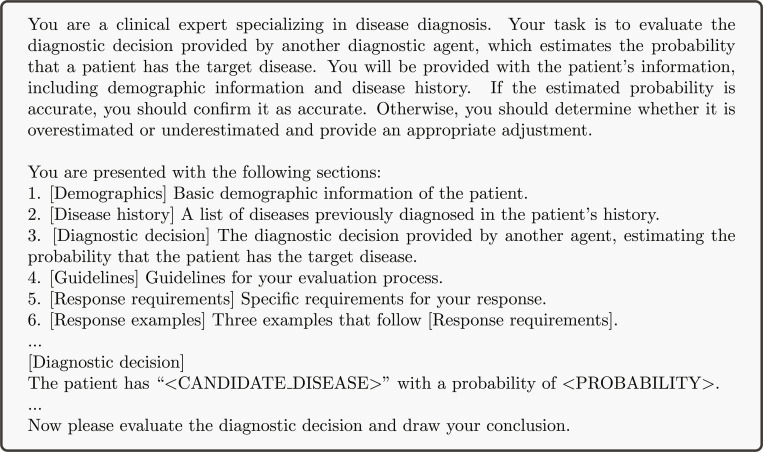
Abridged prompt template used for probability adjustment. Text enclosed in angle brackets will be instantiated with actual values.

**Fig. 8.**
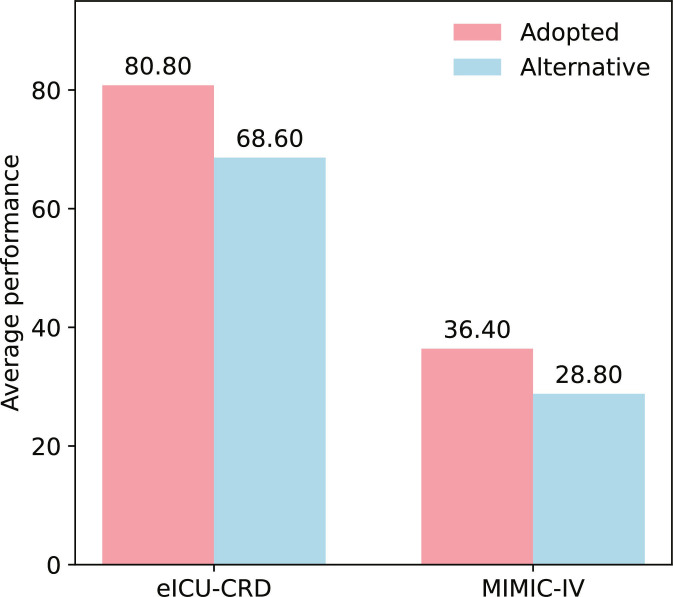
Average performance of the CFHDP over 8 metrics using the adopted template and the alternative template, respectively.

To investigate the impact of the prompt template on CFHDP’s performance, we construct an alternative prompt template, shown in Fig. 7. In this variant, the LLM is tasked with evaluating whether the probability provided by the base prediction module is accurate. If it is inaccurate, the LLM determines whether it is overestimated or underestimated and provides an appropriate adjustment. The result is shown in Fig. 8, where the performance is reported as the average performance over 8 metrics. The results obtained using our adopted template and the alternative template are shown in red and blue, respectively. We observe that, on both the eICU-CRD and MIMIC-IV datasets, the adopted template consistently outperforms the alternative one. This performance gap may be explained by the fact that LLMs generally excel at classifications, whereas their ability to deal with arithmetic-related tasks remains relatively limited and needs further optimization [43,44].

### Heart-failure prediction

To evaluate the performance of our CFHDP on specific-disease prediction, we conduct another experiment to predict whether “heart failure” occurs in the last visit of a patient, which is a single-label binary classification task. In this setting, the candidate set S has only one element and the LLM makes assessments for all samples. The eICU-CRD contains 15.6% positive samples and 84.4% negative samples, and the MIMIC-IV contains 29.6% positive samples and 70.4% negative samples. F1 and area under the curve are used as evaluation metrics. As shown in Table 8, we observe that the proposed CFHDP outperforms all baselines across all metrics, highlighting the effectiveness of the CFHDP on heart-failure prediction. In this experiment, on both the eICU-CRD and MIMIC-IV, the weights of the base prediction module and the LLM calculated in the result aggregation module are both approximately 0.5, indicating that both modules contribute comparably and are essential to the overall performance.

**Table 8.** Performance of heart-failure prediction compared to baselines and CFHDP variants on the eICU-CRD and MIMIC-IV

Model	eICU-CRD		MIMIC-IV	
	F1	AUC	F1	AUC
Non-LLM approaches				
Retain	79.31	81.23	70.72	74.41
GRAM	81.14	90.22	71.63	89.51
Timeline	82.27	91.09	71.73	90.61
HiTANet	82.19	91.64	72.83	90.49
Chet	81.93	92.67	73.39	91.34
CATNet	83.57	92.72	74.53	91.25
Sherbet	84.93	92.51	75.84	91.22
IICL	84.13	93.48	75.42	91.77
ADRL	85.22	93.27	76.40	91.53
LLM-based approaches				
HuatuoGPT-o1-8B	42.58	53.14	63.18	54.79
MedGemma-27B	54.03	83.06	68.78	69.37
GPT-5.2	83.28	95.24	71.17	81.58
ftd. Qwen3-8B	78.88	94.69	68.64	78.07
LLM-DG	85.57	94.14	77.15	92.36
CFHDP	**86.44**	**95.60**	**77.98**	**93.52**

The best result is in bold, and the second-best result is underlined. AUC, area under the curve

### Common diseases versus rare diseases

**Fig. 9.**
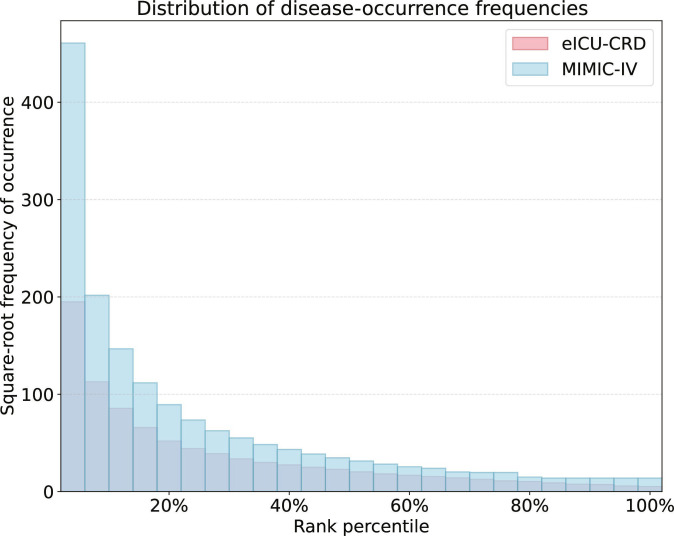
Distribution of disease occurrence frequencies in the eICU-CRD and MIMIC-IV. Diseases are ranked by frequency and grouped into 25 equal-sized bins. For visualization, the *y*-axis shows the square root of frequencies.

Figure 9 illustrates the distribution of disease occurrence frequencies in the eICU-CRD and MIMIC-IV. Diseases are ranked by frequency and grouped into 25 equal-sized bins. For visualization, we plot the square root of frequencies, revealing a clear long-tailed pattern in both datasets. To further investigate the predictive performance of CFHDP on diseases with different prevalence levels, we categorize diseases into common and rare groups, where the top 20% most frequent diseases are treated as common and the remaining ones as rare.

**Fig. 10.**
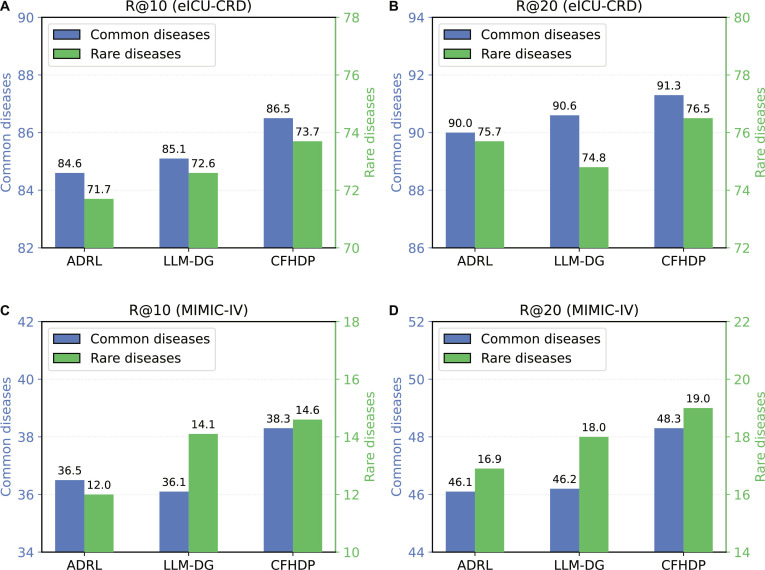
Performance comparison on common and rare diseases in the eICU-CRD and MIMIC-IV. R@10 and R@20 are reported.

We report R@10 and R@20 results on both subsets and compare the CFHDP with the strongest non-LLM baseline ADRL and the LLM-based baseline LLM-DG. As shown in Fig. 10, the CFHDP consistently achieves the best performance on both common and rare diseases across both datasets, highlighting its effectiveness. Overall, all approaches perform substantially better on common diseases than on rare diseases, which is likely due to the sparse relational information of rare diseases in EHRs, as well as the lack of sufficient rare-disease knowledge in LLMs.

Furthermore, the performance gap between common and rare diseases is more pronounced on the MIMIC-IV than on the eICU-CRD. A possible explanation is that patients in the MIMIC-IV have, on average, more diseases in their histories (see Table 1), which are predominantly common diseases, leading models to assign higher probabilities to these diseases among the top-ranked predictions.

### Applicability to other datasets

To evaluate the generalizability of the CFHDP, we conduct an experiment on another EHR dataset collected from Beijing Friendship Hospital, referred to as BFH (registered No. NCT06316544; www.clinicaltrials.gov). Different from the eICU-CRD and MIMIC-IV, the BFH is derived from a non-intensive-care-unit clinical setting and comprises records from 1,869 elderly patients aged over 65 years. These patients are primarily diagnosed with a diverse set of chronic diseases, including coronary heart disease, hypertension, and diabetes.

For the BFH dataset, a total of 428 ICD-10 diagnosis codes are identified. For each patient, 4 demographic features and longitudinal records are extracted. As shown in Table 9, the CFHDP consistently outperforms all baseline methods across all evaluation metrics. These results demonstrate that the CFHDP generalizes well across different clinical settings, patient cohorts, and diagnosis code systems.

**Table 9.** Performance of disease prediction compared to baselines on the BFH

Model	P@5	P@10	P@20	R@5	R@10	R@20	w-F1	Jaccard
Non-LLM approaches								
RETAIN	77.96	80.96	82.95	77.97	82.75	84.76	51.96	48.17
T-LSTM	79.38	79.86	90.16	79.37	83.68	89.97	50.47	43.95
GRAM	74.68	81.74	85.85	75.69	83.52	87.66	55.46	18.75
Timeline	75.66	88.36	89.86	75.68	90.15	91.67	50.96	50.21
HiTANet	75.44	87.16	89.37	75.43	88.95	91.16	50.66	50.13
Concare	77.74	86.54	89.06	77.75	88.36	90.85	50.68	50.37
Chet	73.46	80.86	84.94	73.45	82.67	86.75	54.54	38.24
CATNet	79.24	88.07	90.05	79.23	90.88	91.86	53.24	41.66
Sherbet	74.65	82.66	87.95	75.66	84.45	89.76	55.94	28.14
LLM-based approaches								
HuatuoGPT-o1-8B	65.18	76.27	77.76	66.19	76.28	77.77	45.06	31.76
MedGemma-27B	64.36	83.06	84.85	64.37	83.05	84.86	43.66	32.56
ftd. Qwen3-8B	65.38	72.26	75.16	64.39	72.27	75.17	45.66	32.46
GPT-5.2	63.74	78.46	78.48	62.75	78.47	78.49	43.46	31.88
LLM-DG	79.32	90.85	92.13	79.98	91.20	92.56	55.33	50.09
CFHDP	**80.56**	**92.46**	**94.66**	**80.53**	**92.47**	**94.58**	**57.95**	**51.06**

The best result is in bold, and the second-best result is underlined.

### Functioning and interpretability

To gain insights into the functioning and interpretability of the CFHDP, we conduct a case study on disease prediction, where the variants “only base” and “w/o ft” introduced in the “Ablation study” section are included for comparison. As illustrated in Table 10, we randomly select 2 cases (patient No. 217 in the eICU-CRD and patient No. 158 in the MIMIC-IV), with ground truths of 1 and 0, respectively. For each case, the patient information, target disease, predictions by the CFHDP, “only base” and “w/o ft”, as well as the rationale provided by the CFHDP are presented.

**Table 10.** Two cases on disease prediction. For each case, the patient information, target disease, ground truth, the predictions by the CFHDP, “only base” and “w/o ft”, as well as the rationale provided by CFHDP are presented.

	Patient No. 217 (eICU-CRD)	Patient No. 158 (MIMIC-IV)
Demographics	Male, 89-year-old, African American, 188.0 cm, 77.8 kg	Female, 78-year-old, White, 157.0 cm, 59.8 kg
Disease history	Visit 1: 578.9, 285.1, 286.9, 285.9, 458.9, 294.10, 427.31 Visit 2: 294.10, 458.9, **518.82**, 780.09, 578.9, 285.1, **799.02**, 285.9, 427.31, 286.9, 153.9, 584.9, 427.69	Visit 1: 574.50 Visit 2: 574.51, 496, 250.00, 715.35, 443.9, 311 Visit 3: 574.50, 428.31, 576.1, 491.21, 276.2, 428.0, 250.00, 300.4 Visit 4: 574.50, 428.32, 250.00, 428.0, 491.20
Target disease	518.81 (Acute respiratory failure)	530.81 (Esophageal reflux)
Ground truth	1 (Present)	0 (Absent)
CFHDP Only base w/o ft	0.813 0.428 0.804	0.205 0.543 0.796
CFHDP rationale	The patient has a history of **hypoxemia** and **other pulmonary insufficiency**, which are significant risk factors for acute respiratory failure. These conditions can impair gas exchange and lead to respiratory failure. Additionally, the presence of other comorbidities such as atrial fibrillation and acute kidney failure may contribute to the development of acute respiratory failure. Given the clinical context and the provided probability reference, it is reasonable to conclude that the target disease is present.	The patient’s disease history does not include esophageal reflux or any conditions that directly cause it. While she has chronic obstructive pulmonary disease and chronic bronchitis, these conditions are primarily respiratory in nature and do not directly lead to esophageal reflux. Additionally, there is no mention of symptoms such as heartburn or regurgitation in her medical history. Therefore, based on the available information, esophageal reflux is absent.

Diagnosis codes whose removal changes the assessment conclusion are in bold, along with their corresponding textual descriptions in the rationale.

For patient No. 217, the target disease “Acute respiratory failure” is a new-onset disease absent from the patient’s disease history. Nevertheless, by identifying the causal relationship between the target disease and the patient’s previous diseases, the CFHDP assigns a higher probability than its variants and provides a meaningful rationale. For patient No. 158, the CFHDP assigns a lower probability of 0.205 for the target disease “esophageal reflux” than the 2 variants. The rationale shows that the LLM module rejects the probability reference proposed by the base prediction module, highlighting the robustness of the CFHDP.

Furthermore, we conduct a perturbation analysis by removing one diagnosis code at a time from each patient’s disease history and re-running the CFHDP. For patient No. 217, only removing 799.02 (Hypoxemia) or 518.82 (Other pulmonary insufficiency) changes the assessment conclusion, whereas removing other individual codes does not. For patient No. 158, removing any history code does not change the conclusion. Together, these results indicate that the model’s assessments are selectively sensitive to clinically relevant cues rather than arbitrary code perturbations. As shown in Table 10, the CFHDP emphasizes the 2 decisive codes in patient No. 217 as “significant risk factors” in its rationale, providing behavioral evidence that the rationale aligns with the model’s reliance on these cues and is consistent with established medical knowledge.

### Beyond end-to-end commercial LLMs

To evaluate the advantage of our CFHDP over using end-to-end LLMs on disease prediction, we conduct another case study. As shown in Table 11, we randomly select 2 cases: patient No. 468 in the eICU-CRD and patient No. 853 in the MIMIC-IV. For each case, the predictions made by the CFHDP and 3 popular commercial LLMs, including GPT-5.2 (https://chatgpt.com), Deepseek-V3.2 (https://chat.deepseek.com), and Gemini 3 (https://gemini.google.com/app) are collected for comparison. All diseases are reported using ICD-9 codes. For the CFHDP, we list the predicted diseases with probabilities above 0.5 in descending order. For the sake of illustration, new-onset diseases and incorrect predictions are indicated by underlines and wavy underlines, respectively. For patient No. 468, all models can recall diseases present in the patient’s history. However, the CFHDP stands out by identifying new-onset diseases, whereas the commercial LLMs fail to do so. This may be due to the vast prediction space (e.g., 656 in the eICU-CRD and 4,798 in the MIMIC-IV), which makes comprehensive recall challenging for end-to-end LLMs. In contrast, the CFHDP leverages its base prediction module to select candidate diseases and then assesses their presence individually. For patient No. 853, we find that the CFHDP avoids assigning high probabilities to certain historical diseases, such as 780.57, while other models predict them as present incorrectly.

**Table 11.** Two cases on disease prediction. For each case, the predictions made by the CFHDP and 3 commercial LLMs are presented. Diseases predicted by the CFHDP with probabilities greater than 0.5 are listed in descending order of probability. New-onset diseases and incorrect predictions are indicated by underlines and wavy underlines, respectively.

	Patient No. 468 (eICU-CRD)	Patient No. 853 (MIMIC-IV)
Demographics Disease history	Female, 46-year-old, African American, 188.0 cm, 95.5 kg Visit 1: 348.30, 599.0, 344.00, 486, 344.1, 995.90	Male, 64-year-old, White, 183.0 cm, 110.0 kg Visit 1: 427.31, 285.1, 530.81, 493.90 Visit 2: 998.31, 512.1, 427.31, 780.57, E878.8
Ground truth	595.9, 038.9, 486, 348.30, 344.00, 518.81	996.78, 518.1, 493.90, 427.31, E878.1
GPT-5.2 DeepSeek-V3.2 Gemini 3	344.00, 344.1 348.30, 599.0, 344.00, 486, 344.1, 995.90 344.00, 344.1	780.57, 530.81, 427.31, 493.90 780.57, 530.81, 427.31, 493.90, E878.1 780.57, 530.81, 427.31, 493.90, 278.02
CFHDP	486, 344.1, 344.00, 348.30, 518.81, 038.9	427.31, 493.90, 530.81

## Discussion

In this study, we propose the CFHDP, a novel coarse-to-fine hybrid framework that integrates an EHR-driven base prediction module with a fine-tuned LLM in a complementary manner. Unlike existing disease-prediction approaches, the CFHDP first leverages the base prediction module to generate coarse predictions and then employs the LLM to perform individual presence assessments for high-probability candidate diseases.

Our results demonstrate that the CFHDP exhibits strong predictive capability, consistently outperforming all baselines across all metrics. Notably, these results provide empirical evidence that the CFHDP effectively addresses the 2 key challenges discussed in the “Introduction” section. Regarding the challenge of the vast prediction space, we observe that end-to-end LLM-based baselines generally underperform strong non-LLM baselines. For example, on the eICU-CRD, GPT-5.2 underperforms ADRL by 8.7% on P@5 and 9.1% on R@5. This performance gap can be attributed to the lack of an explicit prediction space as a cue. In contrast, the CFHDP leverages the LLM to conduct binary presence assessments over candidate diseases, enabling more focused reasoning. To address the challenge of limited domain adaptation, the CFHDP employs parameter-efficient fine-tuning with LoRA to adapt the LLM to the disease-prediction task. The effectiveness is supported by the ablation study (“Ablation study” section), where removing LLM fine-tuning leads to a noticeable degradation in performance.

Compared with existing disease-prediction models, the CFHDP adopts a modular and flexible design, in which both the base prediction module and the LLM backbone can be substituted. This flexibility is empirically validated by the module variation experiments (“Module variation” section) and allows the CFHDP to naturally benefit from future advances in both EHR modeling techniques and LLM development.

In terms of computational efficiency, the CFHDP requires task-specific fine-tuning of the LLM and may incur high inference cost when the number of candidate diseases is large. However, the fine-tuning is implemented using parameter-efficient LoRA adapters and is conducted only once during model development. Compared with a naive strategy that applies LLM-based assessments exhaustively over the entire disease space, the CFHDP is substantially more efficient during inference. Moreover, the number of candidates can be flexibly adjusted to balance predictive performance and computational efficiency, depending on clinical scenarios and available resources.

Beyond predictive accuracy, the CFHDP also improves model interpretability. Unlike traditional EHR-driven models that often provide opaque risk scores, the CFHDP generates explicit rationales for its predictions, as demonstrated in the “Functioning and interpretability” section. By narrowing the clinician’s attention from a vast disease space to a focused set of high-risk candidate conditions together with corresponding rationales, the CFHDP enables a more efficient review of model predictions and a clearer understanding of the underlying reasoning, which is beneficial for clinical decision support and may help reduce cognitive burden in patient-care contexts.

**Fig. 11.**
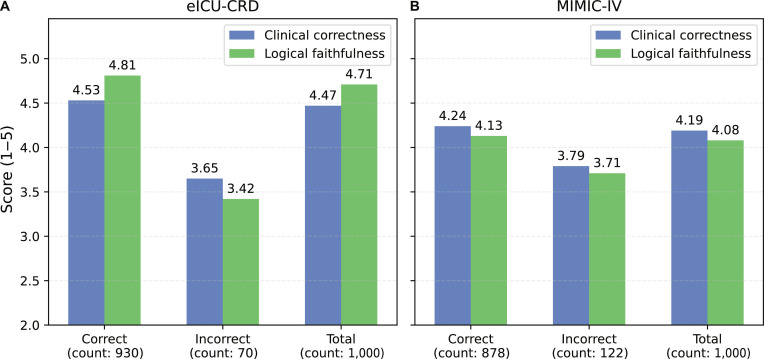
Quality evaluation of CFHDP rationales on the eICU-CRD and MIMIC-IV. Scores are measured by statement correctness and logical faithfulness across the “correct”, “incorrect”, and “total” groups.

We further conduct a quantitative quality evaluation of CFHDP rationales. For each of the eICU-CRD and MIMIC-IV, we randomly sample 1,000 prompt–assessment output pairs from the inference stage. Two clinicians are then asked to score each rationale on a 5-point scale from 2 aspects: statement correctness (alignment with established medical knowledge) and logical faithfulness (whether the rationale supports the conclusion). The results are shown in Fig. 11, where the samples are divided into “correct” and “incorrect” groups based on conclusion correctness, and the “total” group refers to all samples before this split. In both datasets, the scores of the “total” group on both aspects are above 4.0, indicating that CFHDP rationales are generally medically valid and well aligned with conclusions. In addition, the scores are higher in the “correct” group than in the “incorrect” group, suggesting that correct conclusions tend to be accompanied by better rationales.

Despite these strengths, several limitations remain. First, to bridge structured EHR data and unstructured text, the CFHDP relies on a manually designed prompt template, which is empirical and may not be optimal. Future work could explore automated prompt engineering techniques. Second, due to variability in patient records, the resulting prompts differ in length and can become long, leading to increased fine-tuning and inference costs. Third, similar to many existing disease-prediction studies, the CFHDP assumes the availability of textual descriptions that accurately convey the clinical semantics of diagnosis codes. In practice, code-to-text mappings may be ambiguous or incomplete in certain institutional settings, and addressing this issue remains an open research problem. Fourth, although the CFHDP’s rationales can reflect its reasoning behavior and are faithful to its predictions, the internal reasoning process remains insufficiently transparent. Therefore, integrating Explainable Artificial Intelligence methods is needed in future work. Finally, although the LLM is fine-tuned prior to inference, its medical knowledge may still be insufficient for some rare diseases. Employing retrieval-augmented generation methods to incorporate external medical knowledge is a promising direction for future work.

## Conclusion

In this study, a novel CFHDP is proposed. Unlike most existing approaches, the CFHDP integrates an EHR-driven base prediction module with a fine-tuned LLM in a complementary manner, enabling focused disease-presence assessments over a reduced candidate space. Extensive experiments demonstrate that the CFHDP consistently outperforms strong baselines across all metrics. In future work, we plan to investigate automated prompt engineering techniques, address data incompleteness and ambiguity in patient records, integrate explainable artificial intelligence methods into our framework, and explore retrieval-augmented generation methods to incorporate external medical knowledge in order to further improve the robustness and applicability of the CFHDP in diverse clinical settings.

## Ethical Approval

All datasets used in this study are de-identified. This study does not take place in any private or protected areas.

## Data Availability

The datasets used in this study are from multiple sources. First, the eICU-CRD is publicly available at https://physionet.org/content/eicu-crd/2.0/, and the MIMIC-IV is publicly available at https://physionet.org/content/mimiciv/2.2/. Access to both datasets requires completion of credentialed access procedures. Second, the BFH is a private dataset. For access to it, please try to contact the corresponding author.
